# Plague and Trace Metals in Natural Systems

**DOI:** 10.3390/ijerph19169979

**Published:** 2022-08-12

**Authors:** Michael Kosoy, Dean Biggins

**Affiliations:** 1 KB One Health LLC, 3244 Reedgrass Court, Fort Collins, CO 80526, USA; 2 U.S. Geological Survey, Fort Collins Science Center, 2150 Centre Avenue, Building C, Fort Collins, CO 80526, USA

**Keywords:** disease, epidemic, epizootic, mammal, plague, rodent, soil, trace metals, *Yersinia pestis*

## Abstract

All pathogenic organisms are exposed to abiotic influences such as the microclimates and chemical constituents of their environments. Even those pathogens that exist primarily within their hosts or vectors can be influenced directly or indirectly. *Yersinia pestis*, the flea-borne bacterium causing plague, is influenced by climate and its survival in soil suggests a potentially strong influence of soil chemistry. We summarize a series of controlled studies conducted over four decades in Russia by Dr. Evgeny Rotshild and his colleagues that investigated correlations between trace metals in soils, plants, and insects, and the detection of plague in free-ranging small mammals. Trace metal concentrations in plots where plague was detected were up to 20-fold higher or lower compared to associated control plots, and these differences were >2-fold in 22 of 38 comparisons. The results were statistically supported in eight studies involving seven host species in three families and two orders of small mammals. Plague tended to be positively associated with manganese and cobalt, and the plague association was negative for copper, zinc, and molybdenum. In additional studies, these investigators detected similar connections between pasturellosis and concentrations of some chemical elements. A One Health narrative should recognize that the chemistry of soil and water may facilitate or impede epidemics in humans and epizootics in non-human animals.

## 1. Introduction

With the appearance and reappearance of outbreaks of various infectious diseases, there are ongoing efforts to identify ecological factors that play a crucial role in their emergence. The majority of emerging human diseases appear to originate from free-ranging animals. Such diseases are often termed zoonotic. A general descriptor is “diseases in nature communicable to man” [1], a phrase that also identifies a respected international conference for the past 75 years. Although most of the diseases that fall under this category are caused by zoonotic pathogens transmitted by animals, the subtle difference in terminology emphasizes the fact that some of these infectious agents may, at least temporarily, live in an abiotic environment such as soil or water. For example, bacteria causing listeria, leptospirosis, tularemia, yersiniosis, and many others can be carried by mammalian hosts and, in some cases, arthropod vectors but can also live and multiply in either soil or water.

The conditions experienced by these bacteria during their life cycles in the non-host environment are substantially different from the conditions experienced within an animal. Nevertheless, this does not suggest that zoonotic bacteria, which do not persist in soil or water environments for a long period of time, are unaffected by abiotic factors. Rodents (order Rodentia) are important vertebrate hosts of pathogenic bacteria, and many of these animals are evolutionarily adapted to a life in burrows that keep them in close contact with the soil environment, suggesting potential influences of soil chemistry.

Investigations of the influences of climate, especially temperature and precipitation, on disease dynamics seem relatively abundant compared to investigations of influences of soil and water chemistry. However, a potential role of the latter has not been completely ignored. Experiments providing evidence for the effects of chemical elements on the functionality of microorganisms and geochemical influences on the immune status of mammals, including humans, are well documented [2].

A weakness of this area of research is the paucity of reports documenting an association between the concentrations of specific chemical elements in soil or water with dynamics of zoonotic diseases in natural conditions. Existing observations, mostly for plague (caused by the bacterium *Yersinia pestis*), have been conducted in Russia over several decades. The results of these investigations have been published almost exclusively in Russian journals and books and remain quite inaccessible to biologists outside of Russia. This treatise reviews some of those investigations and illustrates a need for a new conceptual approach for investigating the role of environmental factors that may lead to the activation of infectious agents in natural ecosystems.

**Figure 1:**
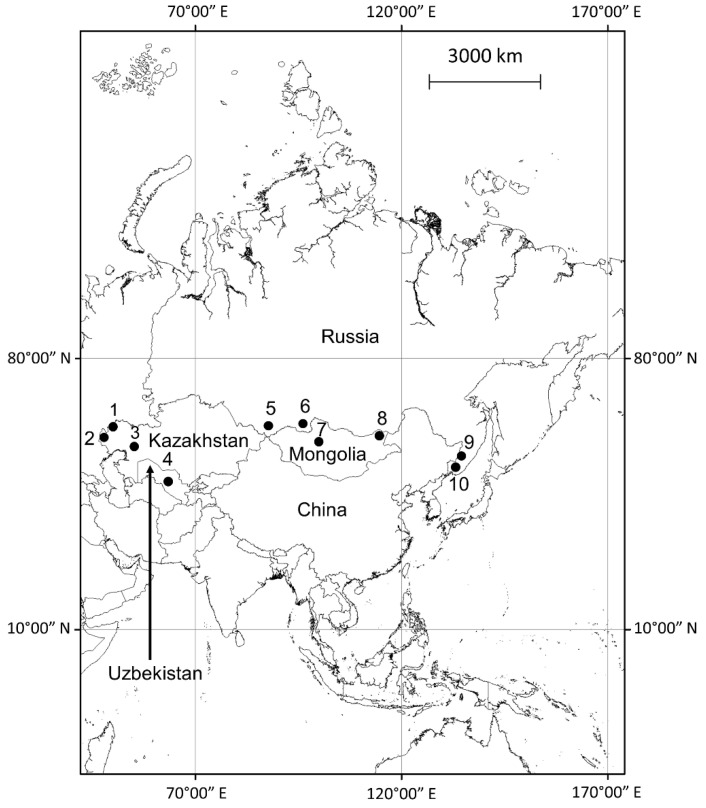
Field sites where Evgeniy Rotshild and his colleagues conducted investigations. Infection, animal species, region: 1—plague, the little souslik (*Spermophilus pygmaeus*), the Caspian Depression; 2—plague, the tamarisk jird (*Meriones tamariscinus*) and the midday jird (M. meridianus), the Caspian Depression; 3—plague, the great gerbil (*Rhombomys opimus*), the low part of Emba River, west Kazakhstan; 4—plague, the great gerbil (*R. opimus*), the Kyzyl-Kum Desert, Uzbekistan; 5—plague, the Pallas’s pika (*Ochotona pallasi*), the Altay Mountains; southern Siberia; 6—plague, the long-tailed ground squirrel (*Urocitellus undulatus*), Tannu-Ola mountains, Tuva, southern Siberia near Mongolian border; 7—plague, the Tarbagan marmot (*Marmota sibirica*), Khangai Mountains, Mongolia; 8—pasteurellosis, Mongolian gazelle (*Procapra gutturosa*), western Mongolia; 9—tick-borne encephalitis, small rodents of the genera *Apodemus* and *Myodes*, far-eastern Russia; 10—hantaviruses, small rodents of the genera *Apodemus* and *Myodes*, far-eastern Russia.

Specifically, the primary focus of this paper is an assessment of the views developed by the prominent Russian ecologist Evgeny Rotshild and colleagues based on >50 investigations of the activity of plague observed under various environmental conditions within the former Soviet Union (Figure 1). Dr. Rotshild emphasized that the described natural phenomena are not restricted to the plague pathogen and not even to bacteria as a category. The summary below of empirical observations made under natural conditions aligns with other available experimental data on the importance of trace metals for infectious microorganisms. These findings might be perceived as a challenge to a dominant paradigm about the continuous circulation of the plague pathogen and some other zoonotic infectious agents among animal hosts, but the various hypothesized mechanisms for disease maintenance are not necessarily mutually exclusive. This discussion is important not only from a strictly theoretical standpoint, but also for the practical significance of forecasting outbreaks of zoonotic infections in general and plague in particular.

## 2. Survival of Plague between Epidemics and Epizootics

The terms epidemic and epizootic can have varied definitions and are often used synonymously. However, epidemic is used more often to refer to severe disease outbreaks in humans, with epizootic referring to similar phenomena in non-human animals. For the purposes of this paper, we consider the primary outcome of a plague epizootic (or epidemic) to be the widespread mortality of the host species within a relatively short time period. After centuries of speculation about potential sources for epidemics of plague in humans, Alexander Yersin not only identified the pathogen *Y. pestis* (*Pasteurella pestis* at that time), but also provided evidence that this pathogen is likely carried by rats [3]. Zabolotnyi demonstrated that non-commensal free-ranging rodents, specifically marmots, can host this bacterium and be a source for human plague [4]. Thus began extensive studies of so-called rodent-borne diseases. Rodent fleas, specifically *Xenopsylla cheopis*, were shown to be an effective vector for the transmission of the plague pathogen among rats [5]. Over the following century, no other zoonotic infection in nature received such intensive attention and research. The Soviet anti-plague system alone heavily invested in supporting the research of thousands of scientists in their investigations of plague in the field and in laboratories.

The commonly accepted view is that *Y. pestis* is hosted by rodents, usually for a limited period of time because of high lethality [6]. Though an enormous amount of information has accumulated as a result of plague investigations, the solutions to many important questions remained obscure. The most intriguing puzzle in the natural history of plague was the question of how *Y. pestis* could be maintained over long periods of only sporadic observable manifestations of this disease in nature. The lack of detection of any visible manifestation of plague can last for many years (even decades) despite the intensive search for *Y. pestis* or its products in animals and arthropods. As an example, plague has reappeared in the northwestern region of Algeria after >50 years without detection [7].

## 3. Plague Pathogens in Soil and Sapronoses

Although live rodents may host *Y. pestis* for a limited period of time before death, their carcasses (and those of fleas) may provide a reservoir for bacterial maintenance in soils. For instance, septic moribund hosts might harbor ≥10^8^ colony-forming units (CFUs) of *Y. pestis* per ml of host blood [8] and total carcasses (with organs) contain additional bacteria. Plague epizootics may kill thousands of rodents (or more) over days to weeks or months, and rodent carcasses accumulate in burrows and runways during outbreaks, thereby depositing huge numbers of *Y. pestis* into the rodent nest soil environment [9], where trace elements may influence *Y. pestis* persistence and dynamics, perhaps even in the absence of continued “rodent-flea” transmission. 

An analysis of Soldatkin and his colleagues [10] was especially influential in challenging the paradigm that *Y. pestis* can exist in nature strictly by a series of continuous “rodent-flea-rodent” passages. These investigators analyzed data collected from numerous field investigations over many years and the results of their analysis raised doubts that plague can interminably persist via the “rodent-flea cycle”. Many other hypotheses have been proposed to determine how the plague pathogen can survive in nature without a continuous transfer of pathogens from one animal host to another.

One reasonable argument is the hypothesis that *Y. pestis* can persist in soil for an undefinable period without an evident pathogenic manifestation and becomes pathogenic under a specific, though unknown, set of ecological triggers. The argument that *Y. pestis* can not only survive but also multiply in soil has a long history. Alexandre Yersin himself claimed that he was able to isolate this organism from soil [3], cited from [11]. Since that time, this concept has remained controversial. In 1960, Mollaret, while investigating the epidemiology of plague in Iranian Kurdistan, provided experimental evidence of the prolonged survival of the bacilli in soil (17 months in sterile soil and seven months in non-sterile soil) while maintaining its virulence [9]. A similar conclusion, along with coining the term “telluric” plague, was drawn by Karimi in 1962 after detecting virulent strains in soil samples collected from burrows of jirds long after any plague epizootic [9].

Studies have demonstrated that *Y. pestis* not only survived in the nest substrate of infected rodents, but also remained able to infect naïve rodents and their fleas [12]. Soil contaminated with *Y. pestis* for 10, 60, 165, 210, and 280 days has been shown to preserve its infectious properties to kill laboratory mice [13]. The Indian Plague Commission also reported the survival of *Y. pestis* in soils [14]. The survival of *Y. pestis* in soil under natural conditions in southwestern regions of the United States for at least 24 days was reported by Eisen et al. [15] in a follow-up study that demonstrated its rare transmission from soil back into mice [16]. These authors emphasized the uncommonness of the phenomenon, but such rare events could be critically important to re-establish cycles with more common modes of transmission.

Another group of related hypotheses concentrates on the supposition that *Y. pestis* can maintain vitality and virulence not in soil as such, but in a close association with soil protozoan organisms [17]. The association of *Y. pestis* with the soil amoeba *Hartmannella rhysodes* was experimentally demonstrated by Nikul’shin et al. [18]. Electron microscopy revealed that, in another free-living protozoan, *Acanthamoeba castellanii*, *Y. pestis* resided within spacious vacuoles intact, which were characterized as being separated from the lysosomal compartment by using lysosomal trackers [19]. Markman et al. [20] conducted environmental genetic surveys and laboratory co-culture infection experiments to assess whether plague bacteria were resistant to digestion by five environmental soil amoeba species. They demonstrated that *Y. pestis* is resistant or transiently resistant to various amoeba species. Additionally, these authors found that the plague bacterium can reside within amoeba structures similar to those found in infected human macrophages, for which *Y. pestis* is a competent pathogen.

The plague bacterium is not the only pathogen that can presumably live and multiply outside animal hosts, specifically in soil. This phenomenon was recognized by the Russian epidemiologist Terskish [21]. Such infections were termed “sapronoses” to distinguish them from zoonoses, diseases which require animal hosts for their circulation [22]. Multiple sources of evidence suggest that most agents causing such diseases are free-living saprophytic microorganisms that absorb and metabolize decomposed organic matter [23]. Many pathogenic bacteria (*Vibrio*, *Yersinia*, *Salmonella*, *Listeria*, *Escherichia*, etc.) exist autonomously in the external environment and infect humans and animals under particular circumstances [24]. Evidence for soil as a source of human pathogens was rigorously reviewed by [25].

## 4. Host–Microbe Interactions and Chemical Elements

In an early study [26], Weinberg demonstrated that soil composition can modulate the incidence and severity of infectious diseases. As possible mechanisms for such an influence, he indicated a suppression or strengthening of host defense by minerals contained in soil, the selective inhibition of pathogens by soil minerals, the availability of minerals to selectively suppress saprophytes to permit the growth of pathogens, and the effect of soil minerals on the survival and growth of intermediate hosts and vectors. In another study [27], Rail suggests that the synthesis of specific factors of virulence in *Y. pestis* depends on the level of specific metallic ions. The varied roles of metallic ions in host–plague interactions are quite similar to the structural and catalytic roles of such ions in free-living macro- and microorganisms. The antimicrobial power of mammalian fluids is depressed by low levels of iron and enhanced by an increase in the iron-binding capacity of a system. The bactericidal power of serum and endotoxin components are affected by the levels of calcium and magnesium. Selenium was shown to play a role in the defense mechanism of animals to certain diseases. Yet, there is sufficient uniqueness of the roles of key metallic ions in many specific host systems for the balance to be tipped in favor of either the host or the plague bacillus through the subtle alteration of the metallic ion environment, specifically within an area endemic for the disease.

This paper is not a comprehensive review of investigations of relations between metallic elements and agents of infectious diseases. We leave that task to others, such as Jerome Nriagu and Eric Skaar [28], who edited “Trace Metals and Infectious Diseases”. The main themes represented in this volume are host–microbe interactions from the perspective of the microbe instead of considering these interactions from the host’s perspective (i.e., metals in environments as risk factors for infectious diseases). Stressing gaps in the latter direction, Ackland ([29], p. 300) stated that: “despite considerable research that is taking place separately on trace metals and infectious pathogens, little is currently known about the interactions between these two key determinants of health…”. Direct observations of the dynamics of trace metals in natural populations of animals in connection with the distribution of infectious agents, either manifested in die-offs or in asymptomatic infections, are few. The simple explanation for this is that it is very difficult to make such observations. Here, we attempt to highlight the relevant research of a team of Russian investigators whose work has not been widely distributed.

## 5. Investigation of Association between Microelements in Natural Environment and Plague Activity in Rodent Reservoirs

### 5.1. Research Methodology Applied by Evgeny Rotshild and Colleagues

Working at the Research Anti-Plague Institute “Microbe” in Saratov, Evgeny Rotshild conducted extensive spatial analyses of the distribution of plague epizootics across main natural foci of this disease in Russia [30]. After continuing his research at the Moscow University Geography Department and later at the Severtsov Institute of Ecology and Evolution, Rotshild was able to access plague research because of his scientific stature and continuous collaboration with scientists of the Russian Anti-plague System. Working closely with scientists investigating plague in the field, Rotshild and his crew identified two kinds of plots: (1) those where plague had been detected either through evidence of rodents dying from the infection or the isolation of *Y. pestis* from animal tissues or their fleas and (2) nearby plots where plague had not been detected over an extended period. Plots were selected on the basis of a shared landscape and ecological characteristics but with varying histories of plague detection. Within each plot, the investigators collected samples of plants commonly consumed by rodents. The main targets for the analysis of environmental sampling were copper (Cu), cobalt (Co), chromium (Cr), iron (Fe), manganese (Mn), molybdenum (Mo), nickel (Ni), vanadium (V), and zinc (Zn).

To compare the concentrations of chemical elements between plague and control plots, Rotshild and his colleagues [31] operated with a metric that they termed “norm”. Basically, this term corresponds to the term “geochemical background”, which is used to distinguish between a “natural background” and “ambient background”. According to the Dictionary of Geological Terms [32]: “In geochemical prospecting, the range in values representing the normal concentration of a given element in a material under investigation such as rock, soil, plants, and water”. Another similar term, “Clarke concentration”, representing the relative abundance of a chemical element, was introduced in the 1930s by the Soviet geochemist Alexander Fersman in honor to the American geochemist Frank Wigglesworth Clarke [33]. Measuring the concentration of particular trace elements in plant or insect samples in “norms” (or Clarkes) instead of using weight units, such as grams, the investigators estimated how much an observed concentration differs from the average concentration of this element in plants of this or related species in a specific study area during the period of investigation. The “norm” for each metal was defined as the mean value for the non-plague plots in each study area. The purpose of using this approach was the attempt to avoid absolute concentrations of elements that varied greatly between study areas and were influenced by different techniques used for measuring concentrations of elements during their investigations in each area. Our assessments used these summary data; the original data were no longer available.

### 5.2. Plague in Ground Squirrels and Trace Metals in Their Putative Plant Diets

The first experience that led Evgeny Rotshild and his colleagues to explore the role of trace metals in the manifestation of plague activity occurred while investigating a plague epizootic among little sousliks (*Spermophilus pygmaeus*). This small ground squirrel belongs to the family Sciuridae and is found from Eastern Europe to Central Asia. The investigation was conducted in the Caspian Depression, which is a flatland region lying much below sea level and encompassing the northern part of the Caspian Sea (Figure 1). The vegetation is relatively sparse and notable for halophytic plants such as sagebrush (genus Artemisia) and some bluegrasses. Another noted geographic pattern specific for this region is a presence of “salt domes” formed by evaporite minerals, mainly sodium chloride. Plague in little susliks within this region has been known since the 1940s, but no plague activity was reported during the following three decades until it was detected again in 1978. Infected little susliks were reported near the middle and lower parts of the Ural River. Sick rodents were found in several clearly separated small areas. The investigators could not find any clear association between the density of rodents and occurrence of plague-infected susliks but assumed a possible connection between the spatial distribution of plague in susliks and presence of the salt domes because such an association had been reported in regard to plague in gerbils in the different parts of the Caspian Depression [34].

The first investigation within this endemic territory was conducted in May 1979. For this investigation, five plots (200–300 m long) were selected where plague was detected during the previous year. Six additional control plots were selected 5–10 km from “plague” plots. As noted above, these control plots were selected based on their landscape and ecological similarity with plots where plague-infected animals were found. Seven species of plants were collected from plague and control plots (*Poa bulbosa*, *Agropyrum desertorum*, *Artemisia lercheana*, *A. pauciflora*, *Ceratocephallus orthoceras*, *Alyssum desertorum*, and *Ceratocarpus turkestanicus*), representing four families (Poaceae, Asteraceae, Ranunculaceae, and Amaranthaceae). The collected plant species are common in this area and frequently used by rodents for food. In total, 28 plant samples from “plague” plots and 43 plant samples from control plots were collected and analyzed to determine the concentrations of five microelements (Mn, Cu, Zn, Mo, and Co) (Table 1 and Table 2). The concentrations of two chemical elements in plants collected from “plague” plots exceeded the “norm” for this area, 2–3 times for manganese and 4–18 times for cobalt [35]. An especially high concentration of cobalt was found in samples of *P. bulbosa* (family Poaceae), a very common food for susliks. In contrast, the concentrations of the other three chemical elements (copper, zinc, and molybdenum) were from 4 to 18 times lower in plants collected from “plague” plots compared to the norm [36]. An especially low concentration of copper was found in plant samples of *P. bulbosa* (Table 1).

In April 1980, plant samples were collected from three additional plots within the Caspian Depression where new cases of plague were reported in little susliks. These samples were investigated along with plants from four control plots selected 4–12 km from the “plague” plots (Table 2). The prevalent plants in all areas were *Anabasis salsa*, *Atriplex cana*, and *E. orientale*. Overall, the analysis of the same chemical elements in plants demonstrated a pattern similar to that of the previous year [35]. Specifically, within the “plague” site, the concentrations of cobalt and manganese were 3 to 10 times higher than those of the control plots depending on the plant species, while the concentration of copper was 2–4 times lower in plague plots than in the control plots [36].

A study of the correlation between plague and metal concentration was conducted on a population of another ground squirrel species, the long-tailed ground squirrel (*Urocitellus undulatus*) [36]. This ground squirrel species is distributed across submontane steppes, plains, meadows, and agricultural land in Southern Siberia and Altai. This investigation of plague was conducted in Tuva Republic, which lies at the geographical center of Asia, in southern Siberia. Plague among the long-tailed ground squirrels was frequently reported within the copper, nickel, and molybdenum ore field on the Tannu-Ola mountains at 1800–2200 m elevation. As a part of plague surveillance, ground squirrels were captured annually from each of the 40 spatially isolated plots. In addition, fleas were collected from the squirrels’ burrows, and all this material was bacteriologically tested for plague. Within this site, cultures of *Y. pestis* were isolated from the ground squirrels and their fleas each year between 1985 and 1989.

During the summers of 1988 and 1989, 58 plant and 12 soil samples were collected to analyze their microelements from eight plots of the ground squirrels affected by plague and from seven control plots where plague had not been reported [36]. Most of the collected plants were legumes (Fabaceae), bluegrass (Poaceae), and sagebrush (*Artemisia*). The collected plant samples were tested for concentrations of seven microelements (Cr, Cu, Fe, Mn, Ni, V, and Zn). The concentrations of some microelements varied substantially between plant groups, by plots and years, rendering comparisons between plague and control plots more difficult [37]. The concentration of chromium was 1.5 times higher and iron was 3 times higher in samples of *Artemisia* collected from plague plots compared to control plots, but not for other plants. The common pattern was 1.5–2 times lower concentrations of vanadium and copper in plague plots than in the control plots (Table 2). Inconsistent patterns were obtained for concentrations of nickel. In plants collected from “plague” plots, the concentration of nickel was either extremely high (4–5 times higher than the norm) or very low (4–7 times lower than the norm). The analysis of soil samples also demonstrated a 1.5 times lower concentration of vanadium and cooper in the samples collected from plague plots compared to control plots. The concentrations of manganese and zinc were not substantially different between plants from plague and control plots [37].

### 5.3. Plague in Jirds and Trace Metals in Their Putative Plant Diets

Plague epizootics in populations of tamarisk jirds (*Meriones tamariscinus*) and midday jirds (*M. meridianus*) were investigated between the Volga and Ural Rivers. Die-offs of the jirds of these two rodent species were registered in eight years during the period from 1966 to 1981. During the epizootic in 1980, almost all Tamarisk jirds died, whereas most midday jirds survived, with more than 50% of them being infected, suggesting higher resistance of the former species to plague. In April 1981 (6–7 months after the epizootic started), plant samples were collected from plots of four types: (1) plots where jirds with *Y. pestis* antibodies were detected at the beginning of epizootic; (2) plots where active epizootics were recorded in the fall of 1980; (3) plots where seropositive animals were found in spring 1982 after the end of active epizootic; and (4) plots where plague in jirds had not been recently detected (Table 2 and Table 3). The investigators wished to examine the relationships between microelements and the dynamics of plague epizootics [29].

The most prevalent plant among the collected samples was cheatgrass (*Bromus tectorum*), a winter annual grass native to the area and the common food of jirds. The concentrations of five chemical elements (Mn, Co, Cu, Zn, and Mo) were assessed in 61 samples of this plant species. The concentrations of copper, zinc, and molybdenum were 1.5 to 3 times lower in plants from plague plots compared to plants from control plots, but the concentrations of manganese and cobalt were higher in plague plots (Table 2 and Table 3).

Unique to this study was the collection of insects (darkling beetles, *Blaps parvicollis,* of the family Tenebrionidae) from the same plots (Table 3). The reason for investigating microelements in beetles is because of the life history of these insects. Their larvae live in soil and feed on plant roots during the warm season of the previous year. Therefore, the investigators assumed that the chemical composition of the chitin shell of adult beetles could retrospectively represent the microelement content in the environment 1–1.5 years before the epizootic. The beetle samples were tested for two elements only (Zn and Co) (Table 2, [36]). Overall, the concentration of zinc was three times lower in beetles from the plague plots compared to those from control plots, whereas the concentration of cobalt was two times higher [31].

When the investigators compared the concentrations of these elements in beetles from the four plots representing different stages of plague activity, they found that the concentration of zinc in all plots where plague started in the fall was >2 times lower than in the control plots and 6–9 times lower in most of them (Table 3). In the control plots, the concentration of zinc was consistent. In contrast, the concentration of cobalt was 3–20 times lower in the control plots compared to the plague plots (Table 3; [36]). The investigators concluded that, in this particular ecological situation, the analysis of microelements in beetles is more informative than the corresponding analysis of plant samples [36].

### 5.4. Plague in Great Gerbils and Trace Metals in Their Putative Plant Diets

Plague epizootics in a population of the great gerbil (*Rhombomys opimus*) were detected along the lower reaches of the Emba River in west Kazakhstan (Figure 1). The Emba river flows through the northern Ust-Urt plateau and reaches the Caspian Sea by a series of shallow lagoons. Plague was active in this region for many years and the spatial distribution of plague in great gerbils in the area was investigated during two periods: 1967–1969 and 1976–1978 [38]. Plague epizootics in great gerbils were detected by culturing bacteria and the presence of specific antibodies within multiple separate plots. An analysis of the influence of geochemical parameters on plague activity was conducted in three plots where plague in gerbils was repeatedly discovered (3–7 times) and in three plots where plague was detected 1–2 times only. In addition, plant samples were collected from two control plots where plague was not detected during the years of investigation. The analysis of the samples of two plant species (*Artemisia lercheana* and *Ceratocarpus turcestanicus*) demonstrated that plants collected from plots with stable plague activity contained 1.5 to 4.0 times less Cu, Zn, and Mo, while 1.5–2 to 3–5 times more Co than samples collected from control plots (Table 2; [36]).

Another investigation of trace metals and plague in the great gerbil was conducted in the central part of the Kyzyl-Kum Desert, located between the rivers Amu Darya and Syr Darya [36]. Based on the results of previous surveillance conducted by the local anti-plague station, samples of plants belonging to either *Aristida* or *Artemisia* species were collected from one plot where plague-infected great gerbils were reported and two plots where plague had not been detected. The results again showed that the concentration of manganese in samples of both plants was 1.5 to 2 times higher in plague plots than in control plots, and the concentration of cobalt was 2–3 times higher in the samples of *Artemisia* and >20 times higher in *Aristida* collected from the plague plots (Table 2). The concentrations of the other metals (Cu, Zn, and Mo) were 1.5 to 3 times lower in plants from the plague plots. An especially high contrast was observed for the concentration of copper in Aristida in plague plots, where it was 12 times lower than in the control plots [36].

### 5.5. Plague in Pikas and Trace Metals in Their Putative Plant Diets

The Pallas’s pika (*Ochotona pallasi*) is the main reservoir of plague in the Altay Mountains, the mountain range of Siberia where its border comes close to China, Mongolia, and Kazakhstan [39]. Chemical analyses of plants in the Altai were conducted in mountainous areas southwest of the Chuiski Valley at elevations of 2000–2500 m above sea level, where plague activity was monitored for many years. For 11 years, from 1975 to 1985, pikas were investigated for plague twice per year in 32 plots in areas of about 0.5 to 1.5 sq. km. Of the 32 plots, eight had plague-infected pikas or their fleas for 5–9 years, 14 plots had such plague detections for 2–4 years, in 5 plots plague was detected for one year only, and plague was never detected in five plots. Plant samples, primarily crested wheat grass (*Agropyron cristatum*), cinquefoil (*Potentilla strigose*), and wild tarragon (*Artemisia dracunculus*), plants commonly consumed by pikas, were collected from 13 plots in summers of 1980, 1981, 1982, and 1985. The plots included those where plague was recently reported in pikas along with control plots where plague was not recently detected.

In 1980, the concentration of manganese in all plants from plague plots was overall higher than in control plots (Table 2), with the following variation among plants: 1.5 times in crested wheat grass, 1.6 times in wild tarragon, and 2.2 times in cinquefoil [39]. The authors also noticed a higher concentration of iron in plants. Specifically, plague was not found in plots with plants that had low concentrations of iron (less than 0.15 of the norm); most plague cases were found on plots with concentrations of iron ranging from 1.6 to 6.0 norms (Table 2).

In contrast, the concentrations of copper and nickel were 2 times lower in most of the plague plots than in control plots. A similar pattern was observed for vanadium. Plague was not detected on plots where the concentrations of nickel and vanadium were higher than 0.75% of the norm. The concentration of zinc was not substantially different between plague and control plots.

### 5.6. Plague in Marmots and Trace Metals in Their Putative Plant Diets

An investigation of plague in Tarbagan marmots (*Marmota sibirica*) was conducted in the southern part of Khangai Mountains in Bayankhongor Province of Mongolia in the fall of 1987 [36]. Plant samples were collected from three plots with active plague epizootics in the marmots and from two control plots. The concentrations of copper in crested wheatgrass collected from plague plots was >20 times lower than in wheatgrass from control plots (Table 2). The concentration of nickel was seven times lower and the concentrations of vanadium and chromium were 1.5–2 times lower in plague plots compared to control plots, whereas the concentrations of manganese, iron, and zinc did not vary substantially between plague and control plots.

### 5.7. Overview of Russian Studies on Plague and Trace Metals

The descriptions above provide evidence for correlations between plague and the relative concentrations of several trace metals. Data from published summaries by study (Table 1 and Table 2) raise questions about sample sizes and numbers of replications that influence statistical inference. The most compelling results are arguably provided by an assessment of patterns over the entire collection of studies. Broad inferences from these patterns were evaluated with simple statistical tests. The emphasis was on repeated measures, comparing each set of plague plots with associated control plots.

Sufficient data were available to separately assess the little suslik study in Kazakhstan (Table 1). The mean concentration over four plant families for each of the five elements was compared for plague and control plots using repeated-measures general linear models (SYSTAT 12.0, Palo Alto, CA, USA), with observations for each plant family serving as individual observations (Table 1). Even with these small sample sizes of summarized data (*n* = four pairs), the consistency of the differences between plague and control plots was emphasized by the *p*-values presented at the bottom of Table 1. For little susliks, plague appeared to be positively associated with manganese and cobalt, and negatively associated with copper, zinc, and molybdenum.

The data for the eight studies summarized in Table 2 were already in the repeated-measures format as categorical comparisons of plague data with paired “norms” within study sites (as described above). If plague has no association with these elements, we assumed that the differences in concentrations of the elements within plague–control pairs of sites would be equally positive and negative. Excluding pairs that had no data or showed no substantial differences (Table 2), we used exact binomial probability tests (Program R; R Core Team 2022, Vienna, Austria) to evaluate the frequencies of remaining differences (*n* ≤ 8 paired comparisons). Missing data for some elements produced small sample sizes that hinder interpretation, but even so, patterns for several elements are unlikely due to random chance. Again, plague seems to be positively associated with manganese and cobalt and negatively associated with copper. There was weaker evidence to support a negative association of plague with zinc and molybdenum.

We emphasize that the analyses of Table 2 reduce the data to the binomial state of positive or negative differences only, ignoring the magnitude of these differences. In fact, plague plots had more than double or less than half the concentrations of trace metals in control plots for 22 of the 38 comparisons (Table 2). An additional noteworthy aspect of these studies is the representation of seven host species in two orders (Rodentia and Lagormorpha) and three families (Ochotonidae, Sciuridae, and Muridae) of mammals. The magnitudes of differences plus the wide representation of host taxa in these studies further underscore the breadth and generality of the influences of trace elements on plague.

We do not know if the associations between elements and plague are independent. If environmental concentrations of individual elements are correlated (positively or negatively), it might be possible for an element with a profound biological influence to produce a detectable apparent influence of another element with which it tends to be found, even if the effect of the second element was inconsequential. In addition, although we have emphasized the possibility that these elements have direct influences on *Y. pestis* in soils, many other indirect influences are possible through effects exerted on fleas and host mammals. It is beyond the scope of this paper to speculate on the many possibilities, but we hope the evidence of general associations provided herein will motivate additional research.

## 6. Other Infections

By conducting investigations in various landscapes, these investigators have affirmed a connection between plague activity and concentrations of some chemical elements. Although most of their investigations have targeted plague activity, some additional studies analyzed other pathogens and have resulted in a similar phenomenon (Table 4). In one study, Evgeny Rotshild and his collaborators reported a correlation between die-offs of Mongolian gazelle (*Procapra gutturosa*) in western Mongolia (Figure 1, point 8), presumably caused by a species of the bacterial genus *Pasteurella*, with the concentration of trace metals (Mn, Fe, Cr, and Ni) in plants eaten by the gazelles (Table 4) [40].

Because pasteurellosis is a bacterial infection similar to plague, a couple more investigations stand out and should be mentioned. The analyses of distributions of two viral infections (tick-borne encephalitis and hantaviruses) in small rodents of several species (*Apodemus agrarius*, *A. peninsulae*, *Myodes rutilus*, and *M. rufocanus*) in far-eastern Russia, close to the Chinese border (Figure 1, points 9 and 10), also demonstrated an association with the distributions of trace metals in plants (Table 4) [41]. These results are particularly interesting not only because of the viral etiology of these infections, but also because they did not have a noticeable impact on animals (in contrast to plague and pasteurellosis). These infections were not based on the presence of epizootics in rodents, but rather on the identification of plots with an elevated prevalence of these viruses in live wild rodents.

## 7. Discussion: Trace Metals and Risk of Zoonotic Diseases

Over more than 30 years, Evgeny Rotshild and his colleagues continuously investigated the spatial distributions of plague activity in association with trace metals in plants. These empirical data have raised questions about the ecology of zoonotic diseases. Traditionally, the emphasis in investigations of zoonotic diseases was on the distribution and ecology of the main vertebrate hosts and arthropod vectors of specific zoonotic pathogens. The distribution of these animal hosts and vectors is clearly affected by various environmental parameters (climate, elevation, landscape, and others). The concentrations of specific chemical elements likely differ in various environments. Less expected is a dramatic influence of small local conditions on a population of one mammalian species that results in either the activation of circulation of pathogens or manifestation of disease in a portion of animals of one population.

Numerous experiments have demonstrated the significant impacts of microelements on the susceptibility of animals to infections. Such studies have allowed the calculation of critical concentrations of microelements in animals for developing infections. In contrast, Evgeny Rotshild and his colleagues did not try to analyze microelement concentration values in their field investigations; in fact, they usually did not report such information in their publications. Instead, they analyzed and reported the concentrations of microelements as deviations in comparison to the concentration observed within a specific area (as they termed the “norm”). Therefore, the most important factor is not an absolute concentration, but a relative concentration of the microelement in a specified area. Their key driving force is ecological stress rather than any particular parameter [42].

Methods for the estimation of metal concentration vary by field study, making it problematic to directly compare results. Thus, we emphasize the patterns in the observed association between geochemical measures and infection activity. Overall, all the studies reviewed are empirical by nature and do not speculate about the mechanisms responsible for the influences of microelements during epidemic manifestations in animal populations. In some studies, the authors reported an association between observed geochemical attributes with particular landscape features, but in other studies such associations remained obscure. The progress made by experimental science in deciphering the effects of trace metals on infectious diseases is fascinating [28]. Field studies conducted in natural habitats deal with extremely complex ecological relationships. Still, investigators working in the field try to take precise measurements of carefully selected attributes. Another approach that could be called “naturalistic” (although the term may not be fashionable for some academic scientists) addresses the ecological situation in general while paying particular attention to changes and variation induced experimentally or happening naturally. The studies conducted by Evgeny Rotshild clearly belong to the latter.

From a theoretical perspective, based on personal communication with Evgeny Rotshild and published papers by Rotshild and his team, it seems that these authors favored a holistic perception developed by the Russian geochemist Vladimir Vernadsky who described the biosphere and, particularly in animals, a close metabolic relationship with the chemical composition of the environment [43,44].

The results summarized herein have been somewhat controversial in Russia. Few have questioned the results themselves, but the presentation of these results without any mechanistic explanation was deemed problematic. Numerous scientific publications generated by Evgeny Rotshild in Russian have never been translated into English except a popularized account published by Science Spectra in 1996 that is not widely available. From the perspective of infection ecology, the transformation of symbiotic microorganisms to pathogens can be investigated through the identification of environmental factors that might trigger the activation of infectious processes [45]. Information about geochemical signals, which activate the pathogenic properties of microorganisms inhabiting soil in natural conditions, remains rudimentary [46].

## 8. Conclusions

The main thesis of Evgeny Rotshild is that environmental changes can lead to the expression of pathogenic activity of some infectious agents. His conclusion emphasizes the critical importance of changes in the geochemistry of soil and plants caused by human activities. Over the last several decades, humans and non-human species alike have experienced wave after wave of new epidemics, some of which have reached the status of pandemics. The One Health narrative connecting the emergence of new infectious diseases with environmental crises is becoming increasingly common. What is often missing is a recognition that rapid changes in the environment, soil and water as basic phenomena may be not only intrinsically destructive but are factors facilitating epidemics. We hope that our reexamination of Rotshild’s data will motivate more intensive research on the relationships between pathogens and chemical attributes of the environment.

## Figures and Tables

**Table 1:** Concentration of trace metals (mg/g dry sample weight; range and mean) in plants col-lected within plots with plague epidemics in little susliks (*Spermophilus pygmaeus*) and in neigh-boring control plots in the Caspian Depression, Kazakhstan, in 1979 (modified from Zhulidov et al., 1981 [35]). *p*-values were derived from repeated-measures comparisons of plague and control mean concentrations with plant families as replicates.

Plant Family		Samples	Mn	Cu	Zn	Mo	Co
*Poaceae*	plague	12	50.8–115.4 (85.5)	0.29–0.54 (0.42)	2.38–3.89 (3.07)	0.48–0.82 (0.63)	2.01–4.12 (3.32)
*Poaceae*	control	24	30.9–45.8 (38.1)	3.45–7.85 (5.45)	15.34–24.73 (18.9)	2.13–3.72 (3.14)	0.12–0.34 (0.21)
*Asteraceae*	plague	28	60.4–108.7 (84.0)	0.84–3.05 (1.84)	3.71–6.85 (4.68)	0.12–0.52 (0.33)	1.74–3.21 (2.40)
*Asteraceae*	control	43	35.1–59.8 (46.6)	4.85–22.12 (14.91)	20.13–39.75 (28.1)	1.75–2.95 (2.17)	0.12–0.71 (0.45)
*Ranunculaceae*	plague	11	77.4–80.9 (79.5)	1.02–1.28 (1.13)	3.21–3.72 (3.39)	0.12–0.24 (0.16)	0.9–1.95 (1.28)
*Ranunculaceae*	control	10	35.4–37.6 (36.7)	6.21–15.31 (11.56)	17.12–26.84 (22.85)	1.34–1.95 (1.6)	0.1–0.51 (0.24)
*Amaranthaceae*	plague	8	170.8–197.3 (183.5)	0.84–0.89 (0.87)	2.71–3.1 (2.91)	0.32–0.39 (0.36)	0.88–0.95 (0.92)
*Amaranthaceae*	control	11	70.2–79.9 (76.3)	3.71–4.32 (3.96)	19.0 0–22.34 (20.40)	1.33–1.54 (1.45)	0.1–0.15 (0.13)
	plague vs. control	*p*-values	0.037	0.042	0.001	0.011	0.046

**Table 2:** Summary of studies of trace metals in plants associated with plague in animals. Values show relative levels of metals in plots where plague was detected compared to paired areas where plague was not detected (controls). *p*-values reflect exact binomial probability tests to evaluate the frequencies of plague-control plot differences, with the null assumption being that differences in concentrations of the elements within plague-control pairs of sites would be equally positive and negative and excluding pairs that had no data or showed no substantial differences (0).

Main Rodent Host/Region	Year	Plots (*n*)	Plants (*n*)	V	Cr	Mn	Fe	Co	Ni	Cu	Zn	Mo
*Spermophilus pygmaeus*/Caspian Depression, Kazakhstan	1979	11	71	nd	nd	2+	nd	2+	nd	2−	2−	2−
*Spermophilus pygmaeus*/Caspian Depression, Kazakhstan	1980	7	13	nd	nd	2+	nd	2+	nd	2−	nd	nd
*Urocitellus undulatus*/Tannu-Ola Mtns., Tuva, Siberia	1988 1989	15	70	1−	1+	0	1+	nd	2+/2−	2−	0	nd
*Meriones tamariscinus* and *M. meridianus*/Caspian Depression, Kazakhstan	1981	61	61			+		1+		2−	2-	1−
*Rhombomys opimus*/western Kazakhstan	1980	6	11			+		2+		2−	2-	1−
*Rhombomys opimus*/Kyzyl-Kum, Uzbekistan	1980	3	6			+		2+		2−	2-	1−
*Ochotona pallasi*/Altay Mtns., southern Siberia	1981 1982 1985	13	79	1−	0	1+	1+	1+	2−	2−	0	nd
*Marmota sibirica*/Khangai Mtns., Mongolia	1987	5	14	1−	1−	0	0	nd	2−	2−	0	nd
			*p*-value	0.13	0.50	0.02	0.25	0.02		0.02	0.06	0.06

0: Concentration of a particular element was not substantially different between plague and control plots (<20%). +: Concentration of a particular element was higher in plague plot than in control plot, but a relative measure was not provided. 1+: Concentration of a particular element was higher in plague plots (20–100%). 2+: Concentration of a particular element was much higher in plague plots (>200%). 1−: Concentration of a particular element was lower in plague plots (20–100%). 2−: Concentration of a particular element was much lower in plague plots (>200%). nd: No data.

**Table 3:** Relative concentrations (ratios of plague plot means to control plot means) of cobalt and zinc in plants and beetles within four plots representing different stages of plague activity in jirds (*Meriones tamariscinus* and *M. meridianus*). Data are modified from Rotshild and Zhulidov, 2000 [31]).

Type of Plot	Beetles (*n*)	Plants (*n*)	Cobalt in Beetles	Cobalt in Plants	Zinc in Beetles	Zinc in Plants
1	9	10	1.48	1.24	3.84	0.69
2	7	15	1.64	1.40	3.23	0.57
3	10	15	2.28	1.04	1.98	0.89
4	11	21	2.92	1.00	1.20	1.00

Types of plots: 1—Plots with earliest records of plague—rodents with antibodies found in spring 1980 (likely plague started in fall). 2—Plots where culture-positive rodents were found, but no seropositive rodents (plague likely started during the 2nd part of 1980). 3—Plots where rodents with antibodies were found in the next year (1981) outside the territory where outbreak started. 4—Plots where plague was not detected in either year (1980 and 1981).

**Table 4:** Concentration of trace metals in plants in places where other animal infections occurred compared to control plots.

Infection/Main Rodent Host/Region	Year	Plots (*n*)	Plants (*n*)	V	Cr	Mn	Fe	Co	Ni	Cu	Zn	Mo
Pasteurellosis/*Procapra gutturosa*/western Mongolia	1983–1984	34	76	nd	2+	1+	1+	2+	2+	0	0	2+
TBE virus/*Apodemus* and *Clethrionomys* spp./Far-Eastern Russia	1982	12	12	2+	0	0	1+	nd	2−	1+	0	nd
Hantaviruses/*Apodemus agrarius*/far eastern Russia	1982	19	19	2+	1+	2+	1+	nd	2−	1−	0	nd

Categories of concentrations for Table 4: 0: Concentration of a particular element was significantly different between plague and control plots (<20%). 1+: Concentration of a particular element was higher in plague plots (20–100%). 2+: Concentration of a particular element was much higher in plague plots (>200%). 1−: Concentration of a particular element was lower in plague plots (20–100%). 2−: Concentration of a particular element was much lower in plague plots (>200%). nd: No data.

## Data Availability

Reviewed data are presented in the tables of this paper. Original data collected by Rotshild and colleagues are no longer available.
